# Strategies for efficient production of recombinant proteins in *Escherichia coli*: alleviating the host burden and enhancing protein activity

**DOI:** 10.1186/s12934-022-01917-y

**Published:** 2022-09-15

**Authors:** Zi-Xu Zhang, Fang-Tong Nong, Yu-Zhou Wang, Chun-Xiao Yan, Yang Gu, Ping Song, Xiao-Man Sun

**Affiliations:** School of Food Science and Pharmaceutical Engineering, Nanjing Normal University, Xuelin Road, Qixia District, Nanjing, People’s Republic of China

**Keywords:** *Escherichia coli*, Recombinant protein, Host burden, Post-translational modification, Inclusion bodies

## Abstract

*Escherichia coli*, one of the most efficient expression hosts for recombinant proteins (RPs), is widely used in chemical, medical, food and other industries. However, conventional expression strains are unable to effectively express proteins with complex structures or toxicity. The key to solving this problem is to alleviate the host burden associated with protein overproduction and to enhance the ability to accurately fold and modify RPs at high expression levels. Here, we summarize the recently developed optimization strategies for the high-level production of RPs from the two aspects of host burden and protein activity. The aim is to maximize the ability of researchers to quickly select an appropriate optimization strategy for improving the production of RPs.

## Introduction

Since the last century, the emergence of recombinant protein (RP) expression systems has revolutionized biotechnology. Excitingly, with the advancement of biotechnology, the yield of RPs has increased from the gram to the kilogram scale, and the range of applications has expanded from traditional food and chemical industries to biopharmaceuticals [1, 2]. For example, it is projected that the industrial enzyme market will grow from USD 6.6 billion in 2021 to USD 9.1 billion by 2026 [3], illustrating the enormous market value and growth potential of RPs. Similarly, a variety of protein drugs have been successfully marketed, including monoclonal antibodies (mAbs), recombinant vaccines, and hormones, demonstrating that RPs already play a significant role in the biopharmaceutical field [4].

Due to its inexpensive fermentation requirements, rapid proliferation ability and stable high-level expression, *Escherichia coli* (hereafter *E. coli*) has become the mainstay of RP expression among prokaryotic expression hosts [5]. As early as the 1970s, *E. coli* was applied in the production of clinical drugs, such as the hormones somatostatin [6] and insulin [7], which were commercialized early on. As a gold standard for expressing RPs, *E. coli* BL21(DE3) and the pET expression system are widely used in research and commercial production. This is primarily attributed to the T7 RNA polymerase (RNAP) from λ prophage in the genome of BL21(DE3), which can specifically recognize the T7 promoter (P_T7_) on the pET plasmid and transcribe at eightfold the speed of the *E. coli* native RNAP [8, 9]. In recent years, several BL21(DE3)-derived strains have been widely used to produce various types of RPs, including C41/C43(DE3) (for the production of membrane proteins) [10], BL21(DE3)-pLysS (for reduction of T7 RNAP expression intensity) [11], BL21Star(DE3) (for improvement of mRNA stability) [12], and SixPack (for codon bias correction) [13]. Such efficient production capacity has given it an unassailable position in structural research, new enzyme mining and industrial production [14, 15].

**Fig. 1:**
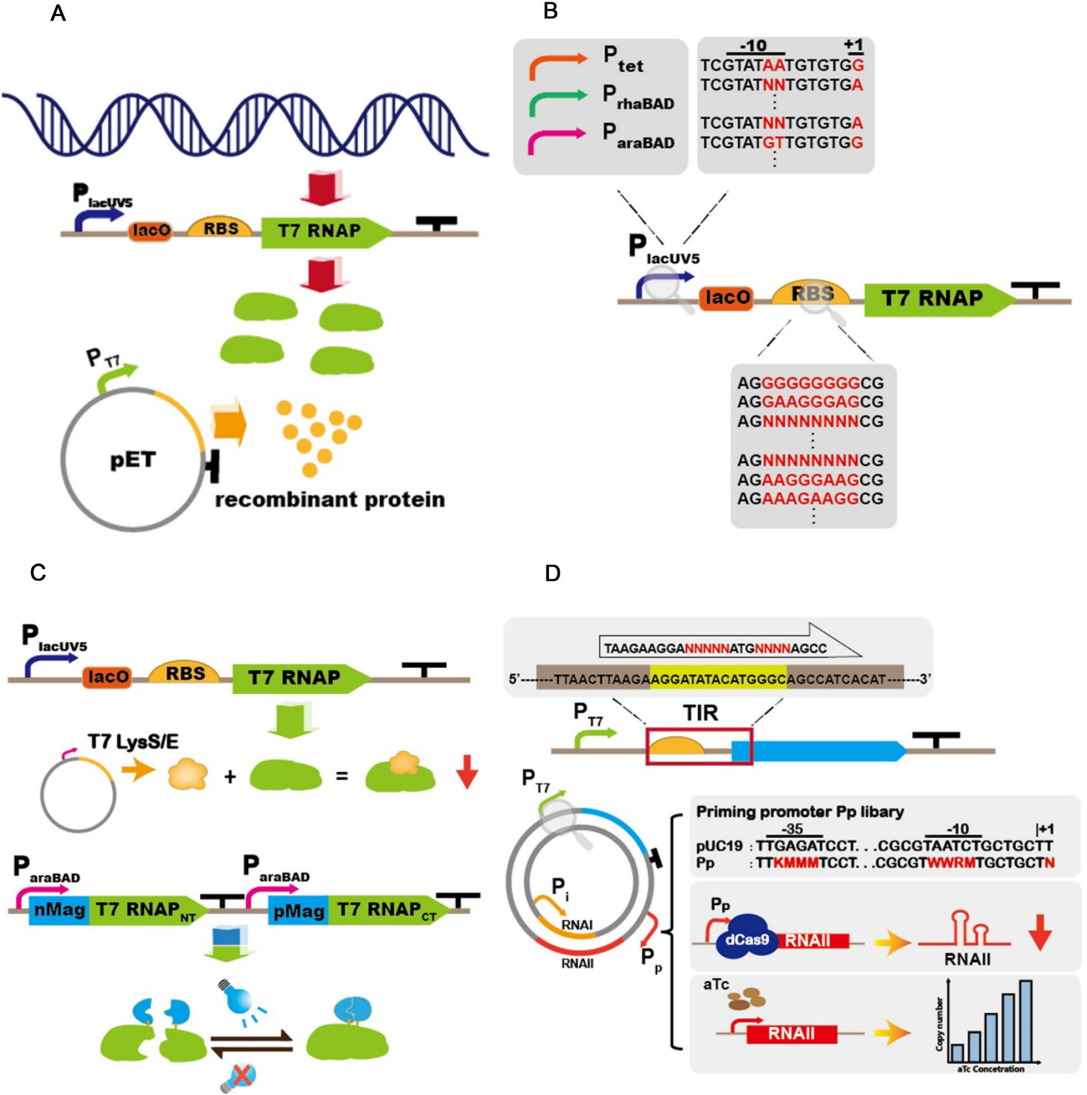
The optimization expression strategies for T7 RNAP and pET plasmids. **A** Illustration of protein expression of recombinant protein genes on pET plasmids. **B** Optimization of T7 RNAP transcription and translation level, including substitutions of different promoters, and mutations in promoter functional region and RBS sequence. **C** regulation of T7 RNAP activity. The conventional approach is to utilize lysozyme or light-induction to regulate. **D** Optimization of pET plasmids based on expression intensity and copy numbers. Among them, the expression intensity was optimized by constructing an ITR library to screen for optimal expression results. The degree of binding of RNA-i to RNA-p determines the replication intensity of the plasmid to control the copy numbers. By constructing a promoter library for RNA-p, replacing the inducible promoter, and using dCas9 to regulate expression intensity, the copy numbers can be controlled

Despite the availability of so many alternative expression systems, there is no guarantee that every type of protein will have a high yield or catalytic/functional activity. The occurrence of these phenomena can be attributed to two main aspects: (i) the host burden caused by the massive production of RPs [16] and (ii) the limited post-translational modification (PTM) capacity and generation of inclusion bodies (IBs) [17]. In fact, any production of RPs, especially toxic proteins, will inevitably compete with the host for resources, which are mainly reflected in the additional DNA replication burden, competition for transcription- and translation-related elements (RNAP, ribosomes, tRNA, and amino acids), and the additional energy and substrates consumed by PTMs [18]. For instance, high-level expression of membrane proteins can lead to the saturation of the Sec translocator-dependent transport pathway, affecting electron transport in the respiratory chain and inhibiting the expression of key enzymes of the tricarboxylic acid cycle [19]. Similarly, glucose dehydrogenase (GDH, an industrial enzyme) leads to significant autolysis of the bacterial cell during the later stages of fermentation [20]. To solve this problem, various means of genetic engineering and synthetic biology have been applied to alleviate host burden, including optimization of the expression intensity of T7 RNAP and pET expression systems (Fig. 1A) [21, 22], as well as balancing or decoupling the cell growth and RP production [23–25]. These optimization strategies effectively relieve or even remove the metabolic burden and increase the capacity of unit cell production. However, when proteins are synthesized at high rates, limited PTMs and molecular chaperones can lead to protein misfolding and the formation of a large number of IBs, affecting the functional activity and solubility of certain proteins. Therefore, the production of highly active RPs is also an important optimization aim, which can be achieved by strengthening or supplementing PTMs, increasing proteolysis and overexpressing suitable molecular chaperones [26]. This review summarizes different classes of optimization strategies developed in recent years from the two main aspects of alleviating host burden and optimizing protein activity, providing a reference for increasing the production of different RPs and discusses the future development direction of related optimization strategies.

## Optimization of target protein expression rate based on the gold standard T7 RNAP platform

When T7 RNAP is sufficiently induced, its powerful transcriptional capacity enables the rapid production of large amounts of mRNA, bringing the yield of RPs to 50% of the total cellular protein in just a few hours [27]. However, a strong production capacity is a double-edged sword, especially in the expression of toxic proteins. Numerous studies have shown that growth inhibition during RP production is mainly attributed to excessively strong gene transcription, and translation further exacerbates the host burden [21, 28, 29]. Therefore, the ability to precisely balance the intensity of RP transcription and translation levels is key to reducing host burden and increasing production. This is usually optimised in two aspects as follows: T7 RNAP and pET plasmid.

## Regulation of the target protein expression rate-T7 RNAP

The easiest way to control the expression intensity of RPs is to regulate the amount and activity of T7 RNAP, which is often achieved by optimizing transcription or translation levels. In the BL21(DE3) genome, the T7 RNAP gene is controlled by the lacUV5 promoter (P_lacUV5_), which is a strongly inducible promoter that ensures rapid expression and accumulation after induction (induced by Isopropyl-beta-d-thiogalactopyranoside (IPTG)) [30]. However, high levels of expression are not compatible with some RPs, especially toxic proteins. Accordingly, many studies increased the production of toxic proteins by reducing the transcript level of T7 RNAP. For example, the membrane protein expression host C41(DE3) was obtained by stress screening, while the autolysin expression host BL21(DE3-lac1G) was constructed by recombining P_lacUV5_ with P_lac_ sequences [10, 20, 31]. Furthermore, the P_lacUV5_ is independent of CRP, which makes it leakier than P_lac_ [32]. Replacing the promoter of T7 RNAP with other kinds of inducible promoters is an effective way to regulate transcription levels and reduce leakage (Fig. 1B). Du et al. [32] tested the effects of three inducible promoters (P_araBAD_, P_rhaBAD_ and P_tet_) on the transcriptional intensity and leaky expression of T7 RNAP, respectively. It was found that all three promoters were suitable for prolonged fermentation of toxic proteins, whereby P_rhaBAD_ and P_tet_ were able to regulate T7 RNAP transcription more rigorously, providing additional options for the expression of various RPs, especially toxic proteins. Similarly, enhancing the ability to block proteins is also an effective way to reduce leaky expression. In addition to the conversion of PlacUV5 to Plac, the study found that the lac repressor gene (lacI) was also mutated (V192F, referred to as mLacI hereafter) in the membrane protein expression host (C41/C43(DE3)) [33]. Excitingly, mLacI can specifically bind to the lac operator site, but the blocking effect cannot be removed by the addition of IPTG. Based on this phenomenon, Kim et al. [31] developed an anti-leakage expression system for the overproduction of membrane proteins. Among them, mLacI expression is regulated by the rhamnose inducible promoter P_rhaBAD_. When trace amounts of L-rhamnose were added, T7 RNAP leakage expression could be inhibited during host growth, reducing growth burden. With the increasing concentration of L-rhamnose, mLacI is abundantly produced and thus reduces the transcription intensity of T7 RNAP, even in the presence of IPTG. This approach makes it possible to control the rate of protein production.

Unlike the transcriptional level, which is controlled by the promoter and RNAP, the strength of translation is mainly determined by the nucleotide sequence and arrangement of the ribosome binding site (RBS) (Fig. 1B). Liang et al. [34] designed 10 RBS sequences with different expression intensities for expressing T7RNAP using an RBS calculator, which was successfully implemented in five Gram-negative and one Gram-positive bacteria. To further extend the regulatory range, Li et al. [35] constructed a more extensive RBS library of T7 RNAP using CRISPR/Cas9 and cytosine base editor, with expression levels ranging from 28 to 220% of the wild-type strain. Using this library, the authors obtained customized hosts for eight difficult-to-express proteins in just three days. The tested model RPs included an autolytic protein, membrane protein, antimicrobial peptide, and insoluble protein, while the production of the industrial enzyme GDH was increased 298-fold. These results show that optimizing the expression intensity of T7 RNAP can effectively improve the RP production, and regulation of the translational level makes it easier to construct screening libraries and rapidly obtain optimized hosts for individual RPs.

Since it is an enzyme, the catalytic activity of T7 RNAP is also a key factor affecting the rate and efficiency of transcription. Mutations of key amino acid residues in T7 RNAP are one of the most effective methods to tune its activity, whose mechanisms are divided into two categories: weakening the binding ability to P_T7_ or generating code-shifting mutations to reduce the catalytic activity [36–38]. For example, Baumgarten et al. [37] found a single amino acid mutation (A102D) of T7 RNAP in the membrane protein expression host Mt56(DE3), which reduced the ability to bind to the P_T7_ and decreased the RP production rate. In addition, the addition of T7 RNAP inhibitors is also a way to effectively regulate T7 RNAP activity, and various derivative hosts including BL21(DE3)-pLysS, BL21(DE3)-pLysE, and Lemo21(DE3) have been developed based on this principle [39–41] (Fig. 1C). With the development of synthetic biology, researchers hope to change the strength of T7 RNAP activity in logic gates to precisely and dynamically regulate the process of growth and production. A variety of T7 RNAP expression systems regulated by light induction have been developed successively, achieving dynamic regulation of RP production [42–44]. For example, the Opto-T7RNAPs system splits the T7RNAP into two fragments and expresses them in tandem with a light-sensitive dimerization domain. When the fragments are expressed and irradiated by the light of a specific wavelength, T7 RNAP can resume its transcriptional activity, with up to 80-fold change in activity between blue light and darkness [43]. Regrettably, these studies have only been validated with fluorescent proteins or lycopene, and have not been applied to RP production.

## Regulation of the target protein expression rate-pET plasmid

Another key factor affecting the expression rate of RPs depends on the combination of different elements on the pET plasmid, including sequences of relevant functional regions near P_T7_ (-35/-10 region, translation initiation region (TIR) and operator sequence) and replicon [45]. As the core region of the pET plasmid, various functional regions near the P_T7_ determine the rigor of basal expression before induction and the appropriate transcription rate after induction.

To reduce the host burden of leaky expression, several more rigorous inducible systems have been combined with P_T7_ to increase the yield of toxic or structurally complex proteins, such as the cumate operator [46], inducible translational ON orthogonal riboswitch [47], and temperature-regulated self-induction [48]. After solving the leaky expression problem, an urgent task is to quickly screen the appropriate expression intensity of various RPs. In contrast to complex genomic manipulations, the combination of degenerate primers and MEGAWHOP PCR or enzymatic digestion and ligation allows rapid access to very large libraries of various functional sequences, including promoter mutation and TIR libraries [22, 49–51]. It is worth noting that the optimal promoter-TIR combination will not necessarily give the best results (Fig. 1D). For example, the optimal combination yielded a 131-fold increase in the expression of superfolder green fluorescent protein (sfGFP), while the highest yield was achieved after single-factor optimization (TIR) of the expression of DNA glycosylase Neil3, with a threefold increase, and combinatorial optimization produced only a twofold increase [22]. Therefore, the use of resistance markers to flexibly screen the expression levels of RPs is expected to become a faster and more accurate library screening tool, especially when multiple libraries are combined [52].

Replicons, genetic elements that replicate as autonomous units, determine the copy numbers of vectors and compatibility with other plasmids. As many expression units reside in each cell, it is logical to assume that a high plasmid dosage results in higher production of RPs [45]. However, this view does not apply to all RPs, as high copy numbers can contribute to rapid accumulation of large amounts of mRNA and RPs, resulting in increased host burden. It was found that each additional plasmid molecule in the host cell increases the metabolic burden by 0.063% [53]. Therefore, an appropriate copy number can provide a balance between growth and production. Generally, replicon replacement is a preferred method for regulating copy numbers [38, 54], with choices ranging from high-copy-number replicons (pUC series, 500–700 copies [55]) to low-copy-number replicons (pSC101, < 5 copies [56]). However, this permanent adjustment of copy numbers makes it difficult to balance the host burden of high copy numbers or low production due to insufficient plasmid copies. Recently, this challenge has been overcome by the dynamic copy number regulation system, which works by regulating key genes of the plasmid replication machinery (priming RNA (RNA-p) and inhibitory RNA (RNA-i)). The degree of binding of RNA-i to RNA-p determines the replication intensity of the plasmid to control the copy numbers. Using inducer-based RNA-p/i promoter libraries, CRISPRi and inducer regulation (Fig. 1D), multiple replicons based on ColE1 can achieve controlled regulation of copy numbers during RP production [53, 57]. For example, Rouches et al. constructed a pUC19 plasmid library spanning 1194 mutants to achieve copy number variations between 1 and 800, thereby optimizing the violacein synthesis pathway and the efficiency of CRISPRi [53]. The appearance of dynamic copy number regulation systems has changed the traditional handling of gene copy numbers, providing a powerful tool to reduce the host burden and improve RP production.

## Dual optimization of growth and production—balancing and decoupling

During the exponential growth phase, the content of RNAPs, ribosomes and various essential proteins is generally constant [58]. Coincidentally, induction of RP expression is usually done in the mid-exponential phase, but rapid transcription and translation can lead to an uneven distribution of host resources and thus affect growth [59]. Ceroni et al. [60] developed a burden monitor that allows real-time detection of the host burden through changes in green fluorescence intensity (GFP integrated into the λ locus). It was found that the expression intensity of RPs and the molecular weight was proportional to the host burden in MG1655 and DH10β, with the highest reduction of fluorescence intensity reaching more than 90%. At the same time, there was a significant decrease in RP production under high burden conditions. Therefore, another key to improving RP production is to achieve the dual optimization of growth and production, which is best solved by balancing the allocation of resources or removing the interference between the two fermentation stages.

### Balancing cell growth and recombinant protein production

No matter how the production rate is optimized, the RPs will compete for the host nutritional resources, affecting normal growth. Exogenous supplementation can effectively compensate for the nutrients consumed during RP production. Depending on the consumption during the production of pramlintide, some amino acids are categorized as growth-promoting (GP1, including serine, aspartic acid, glutamic acid, threonine and proline) and protein production promoting (GP2, including cysteine, methionine, leucine and alanine) [24]. The combination of 5 mM GP1 at inoculation with 2.5 mM GP1 and GP2 after 6 h in fermentation was the most economical and effective, resulting in a 40% increase of pramlintide production (protein concentration of 3.09 ± 0.12 g/L). In addition, this strategy was also applied to the production of granulocyte colony-stimulating factors.

For the host, reducing unnecessary energy expenditure or blocking byproduct formation can effectively alleviate the burden associated with RP production. The accumulation of acetate is an important factor in the RP production, since it inhibits cell growth and protein synthesis [59]. Blocking the phosphotransferase system (PTS) can effectively reduce the rate of glucose uptake and decrease the production of acetate, which has been applied to increase the production of enhanced GFP (eGFP) [61], vaccines [62], and glutamate dehydrogenase [63]. In addition, knocking out flagellar formation-related genes can reduce energy consumption in *E. coli*. Jae et al. [55] further knocked down the major flagellar regulator (FlhC) in a PTS-blocked strain, which increased the ATP pool and NADPH/NADP^+^ ratio. These strategies demonstrate that it is feasible to redistribute energy metabolism and reduce by-product formation for the increased RP production.

In addition to the host burden caused by competition for resources, the RP production often triggers a cellular stress response (CSR). Therefore, blocking the emergence of CSR can prevent the down-regulation of a large number of growth-related genes and alleviate the negative effects of CSR on the host [64]. Sharma et al. [64] compared the transcriptomes of cultures of different RPs and selected a series of up-regulated genes for knockout. The results showed that the double knockout mutant BW25113ΔelaA + ΔcysW (DKO) had the highest activity in asparaginase production with 70.3 units/ml. To further unravel the mechanisms involved in CSR mitigation by the DKO strain, Guleria et al. [65] used the strain to overexpress the Rubella E1 gene and performed a transcriptome analysis. Compared to the wild type, down-regulation of multiple genes related to growth-critical processes was suppressed in the DKO strain, including translation, transcription, RNA and ribosome biogenesis, transport, energy metabolism and other catabolic processes. It suggests that the host burden caused by RPs can be effectively mitigated by blocking CSR, which has the potential to serve as a chassis cell to develop an efficient platform for recombinant protein production.

In general, the native genes encoding most heterologous RPs have rare codons, which often affect their translation and folding rate [66]. Two strategies can be applied to alleviate the host burden: heterologous gene codon optimization and supplementation of rare tRNAs. The former not only requires significant experimental resources, but also results in heavy competition for the internal tRNA pool, placing a heavier burden on the host [67]. Conversely, the appropriate introduction of rare codons can improve the yield and solubility of RPs and reduce the host burden [68, 69]. Accordingly, the overexpression of rare tRNAs is a more economical means of optimization. A variety of commercial expression strains, including the Rosetta™(DE3) series and BL21-CodonPlus(DE3), have been developed based on this principle [45]. Unlike the two commercial strains, the newly developed expression host SixPack [13] integrates six of the least abundant tRNA genes into the BL21(DE3) chromosome behind a ribosomal manipulator for expression. This not only relieves the burden of plasmid-based tRNA expression, but also regulates the expression intensity of rare tRNAs through ribosomes, avoiding the waste of resources. This host has been demonstrated to outperform BL21(DE3) and Rosetta2(DE3) in the expression of RPs from eight different origins.

### Decoupling cell growth and recombinant protein production

The mechanisms inducing host burdens vary depending on the class of RPs, and a more simplistic approach would be to decouple the cell growth from RP production, effectively reducing the difficulty of resource allocation. In the first stage, the host cells are cultured at a normal growth rate without competition from RP production. Once the culture has reached the stable stage, growth will be stopped and RP production induced so that most of the resources are used for product synthesis. This two-stage fermentation process has been successfully applied to RP production [70].

The auto-induction system is a decoupling method often applied in industrial production. Traditional auto-induction media are usually supplemented with glucose, lactose, or glycerol. When glucose is present, it inhibits the uptake of lactose by the bacterium and prevents RP production. After glucose is exhausted, lactose is transported into the cells to induce RP production [71]. To further expand the range of applications and reduce leaky expression, several types of auto-induction systems have been developed, based on principles such as quorum sensing [72], phosphate induction [73], or molecular chaperones that unblock catabolite repression [74, 75]. Notably, the phosphate-based auto-induction system can be used under different culture conditions, including 384-well plates, shake flasks and bioreactors [69]. Melgar et al. [76] combined this system with lysozyme and DNA/RNA endonuclease to achieve auto-induction and autolysis, allowing the release of more than 90% of the protein and facilitating its application in industrial production.

**Fig. 2:**
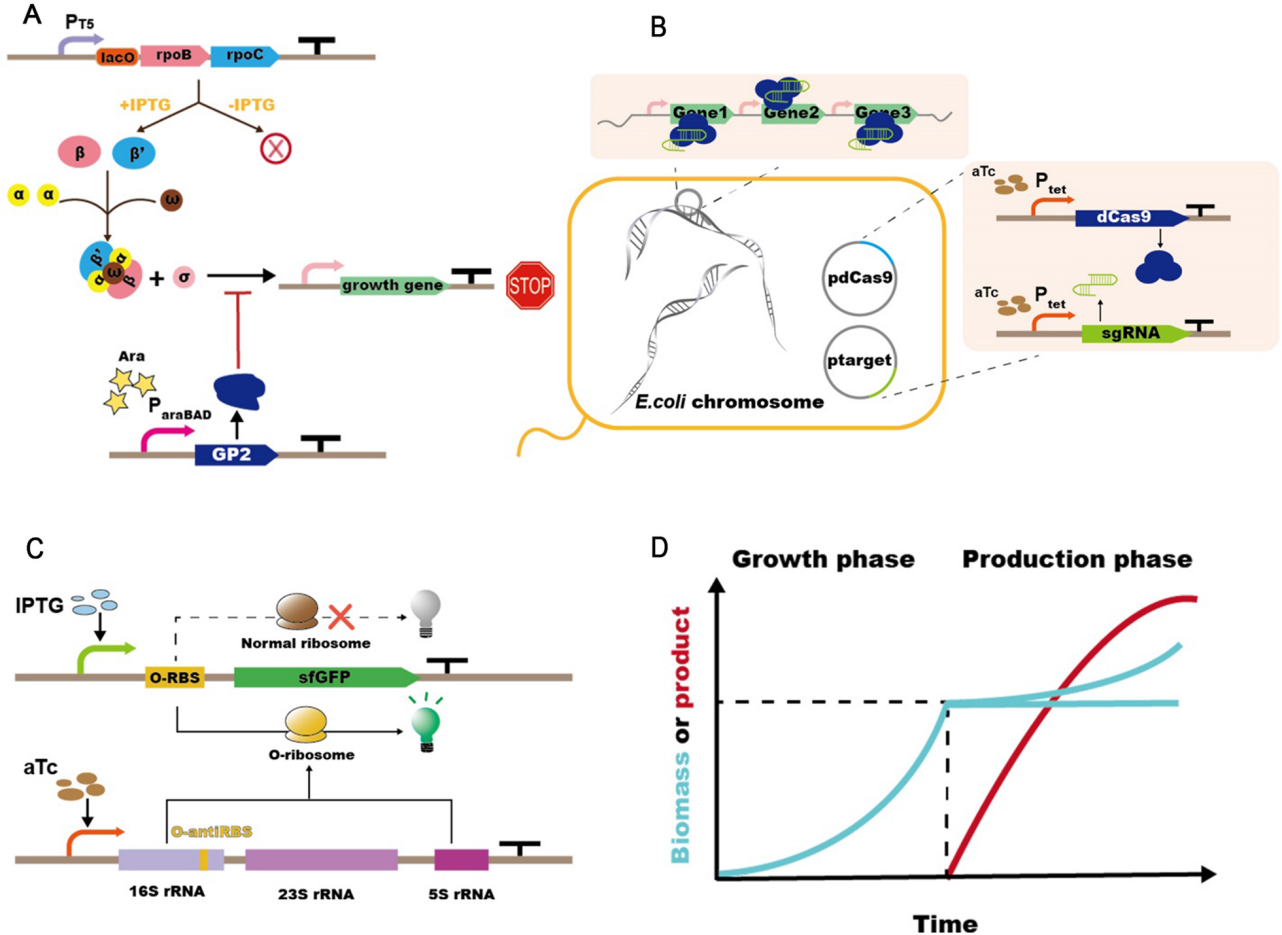
The optimization expression strategies for decoupling the cell growth and RP production. **A** Manipulating the expression of RNAP subunits (β and β') or inhibiting RNAP activity by RNA polymerase inhibitor GP2 to prevent transcription of endogenous growth genes. **B** Inhibition of growth-related gene expression using CRISPRi. **C** Reducing competition for host ribosome using orthologous ribosome (O-ribosome) to specifically translate target proteins. **D** The uncoupling strategy allows to clearly divide an RP production process into two phases, namely the growth phase and the production phase. This allows resources to be used for RP production during fermentation

However, auto-induction systems cannot achieve growth arrest during production, and interrupting cell growth can more efficiently allocate resources to RP production, which is often achieved by inhibiting or blocking the expression of growth-critical genes. A variety of decoupling strategies have been applied to RP production by controlling or inhibiting the expression of endogenous RNAP (Fig. 2A) [25, 77, 78]. Excitingly, blocking the expression of endogenous RNAP improves the efficiency of the insertion of non-canonical amino acids (ncAA) at specific sites, expanding the application range of this strategy [79]. Similarly, blocking the normal replication of chromosomes can also achieve growth arrest. Kasari et al. [80] added serine recombinase recognition sites at both ends of the replication start (oriC) of the chromosome and blocked normal DNA replication by temperature-induced expression of serine recombinase, which resulted in a fivefold increase in the product yield. However, this approach completely blocks the normal growth of the host and cannot achieve a dynamic balance between growth and production. By contrast, inhibition of growth-related proteins (DNA replication, or nucleotide synthesis-related proteins) using CRISPRi can dynamically regulate the growth state (Fig. 2B) [81]. Li et al. [82] constructed a sgRNA library targeting growth-related genes, and 1332 different sgRNAs were screened to reduce host growth and increase GFP accumulation. Among them, GFP production increased more than fivefold when *sibB*/*ibsB* was inhibited.

In fact, the fundamental purpose of decoupling growth and production is to make the best use of the host resources. If a series of orthologous elements are utilized to prevent RP production from depleting key growth resources, the goal of alleviating the host burden can be achieved. Because of the universality and complexity of the cellular translation machinery, there is no unique ribosome in *E. coli* that recognizes specific mRNAs to achieve orthogonal translation [83]. Interaction between RBS and 16S rRNA in the ribosomal subunit is a key regulatory step in the recognition and initiation of translation (Fig. 2C). Darlington et al. [83] evaluated the feasibility of developing orthogonal translation systems development by modeling, further customizing 16S rRNA to successfully develop a more efficient orthologous ribosome (o-ribosome). When no orthologous mRNA is present in the host, the o-ribosome can still translate the endogenous mRNA. With increasing expression of the orthologous mRNA, the o-ribosome recognizes and translates it, preventing this mRNA from occupying the host ribosome and interfering with normal metabolism, which is especially useful in the expression of toxic proteins. However, the o-ribosome is defective and produces proteins with a tenfold lower capacity than that of the natural ribosome. To solve this problem, various optimization strategies have been applied to improve the orthogonal translation system in recent years [84, 85]. Among them, Liu et al. [84] utilized phage-assisted continuous evolution technology for rapid optimization of 16S rRNA by screening pressure. After multiple rounds of directed evolution, the mutant o-ribosome achieved faster translation, resulting in 6.3-fold higher RP production than the wild-type. Most importantly, this ribosome can introduce ncAAs into the protein with high efficiency, which is 9.08-fold higher than that of the native ribosome, improving the application of orthogonal translation systems in RP production. In brief, whether it is to inhibit or block the expression of growth-essential genes or to use o-ribosomes to express RPs, the aim is to ensure normal growth of the host during the growth phase (Fig. 2D).

## Optimizing protein activity—another key to the production

In addition to ensuring the quantity of RPs, the functional activity of the protein at high yields is also a key focus of RP production. When the expression rate or quantity of RPs exceeds the capacity of the host cell, it will result in a large number of proteins that misfold and aggregate, eventually producing IBs [17]. This phenomenon has greatly hindered the use of *E. coli* in various fields, especially the expression of protein-based drugs. The key reason for the generation of IBs is the limited PTM capacity and folding efficiency, which are the top priorities for optimizing the functional activity of RPs.

### Enhancement of post-translational modifications

Most proteins with complex structures contain multiple disulfide bonds (DSBs) that maintain their normal conformation, including insulin [7] and epidermal growth factor [86]. As an oxidative process, the natural DSB formation is completed in the periplasmic space of *E. coli* and not in the reductive environment of the cytoplasm, which requires the protein to be localized and translocated to the appropriate location for modification [87]. The common protein translocation pathways are divided into three main categories: SecB-dependent, SRP-mediated and TAT translocation pathways [88]. Among them, SecB-dependent and SRP-mediated pathways both complete the translocation process by binding to SecA, and genetic fusion of signal peptides to RPs can enable them to utilize these pathways to translocate. Commonly used signal peptides include pelB, OmpA and DsbA [89, 90], but each signal peptide triggers a different mechanism that greatly affects the effectiveness of RP transport. In contrast to SRP-mediated DsbA, SecB-dependent OmpA drives the synthesis of endogenous secreted and membrane proteins, preventing Sec translocator saturation [89]. In recent years, the TAT translocation pathway has attracted the interest of researchers due to its natural "quality control" system, which can prioritize the output of correctly folded proteins [91]. The "TatExpress" strain was successfully developed and applied for the gram-level production of human growth hormone, proving its great potential [92]. In addition to the above translocation pathways, a signal peptide based on the N-terminal sequence of penicillin-binding protein 2 (PBP2) was shown to anchor the fusion protein to the cytoplasmic membrane. Interestingly, the high expression of PBP2 affects morphological changes in *E. coli* (rods to spheres) and interacts with lysis transglycosylase leading to host lysis [93]. This phenomenon has the potential to be developed into a self-cleaving transport system for rapidly accumulating RPs production.

**Fig. 3:**
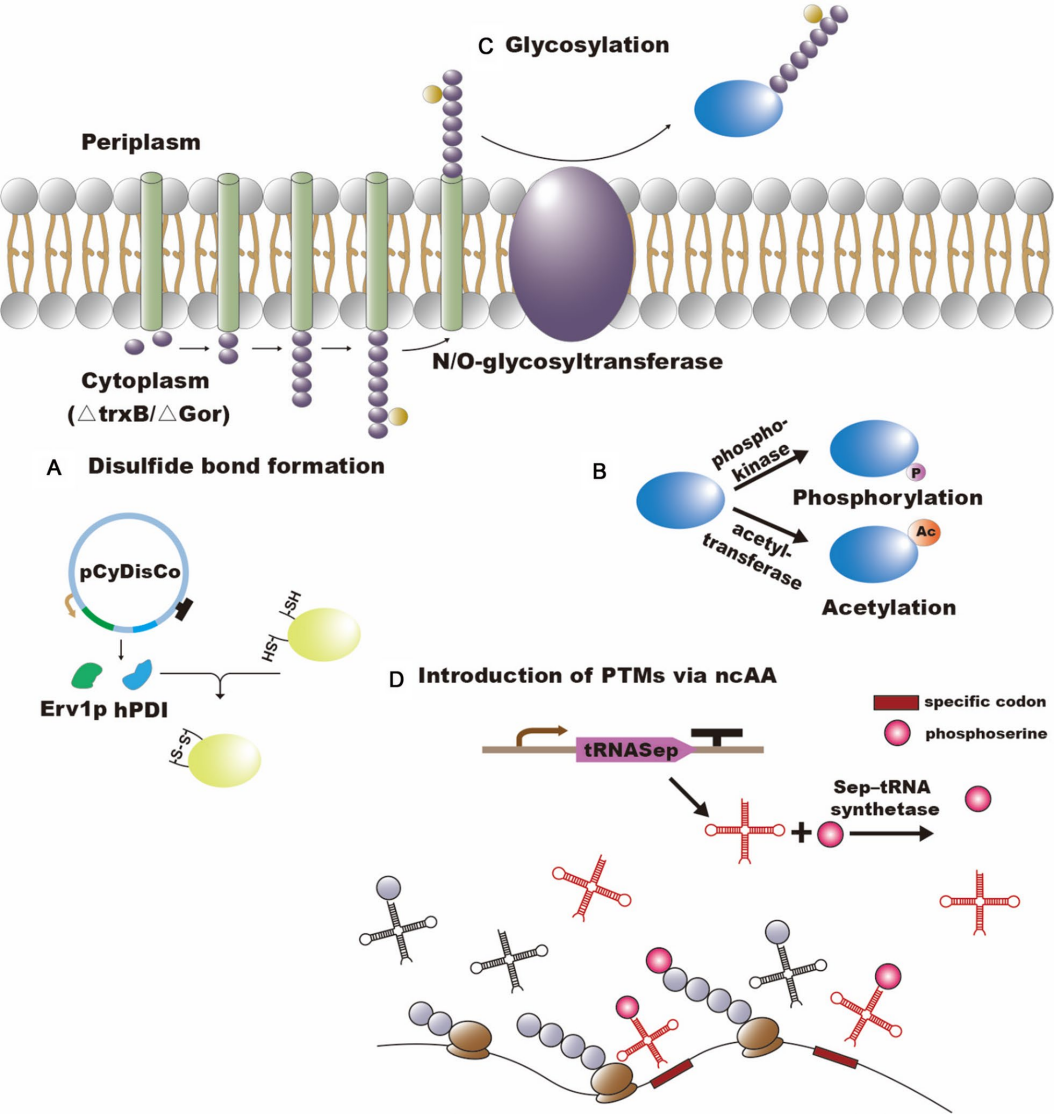
The optimization strategies to enhance PTMs. **A** Principle of disulfide bond formation in the cytoplasm using the CyDisCo system. **B** Modification process of phosphorylation and acetylation. P: phosphonate; AC: acetyl. **C** Modification process of glycosylation by overexpression of a heterologous N/O-glycosylase. **D** Introduction of PTMs via ncAA. The figure shows the principle of phosphoserine introduction

Compared to the narrow periplasmic space, the cytoplasm has enough space to accomplish more protein folding and increase productivity. By blocking the natural reduction pathway in a Δ*gor*/Δ*trxB* strain, the reductive cytoplasmic environment becomes oxidative, which facilitates the formation of DSBs [94]. The earliest commercial DSB-forming *E. coli* strain, Origami from Novagen, was developed based on this principle. By overexpressing sulfhydryl oxidase from the yeast mitochondria and disulfide bond isomerase from human cells, a host called CyDisCo was developed for the production of RPs with high DSB content, and was able to produce even perlecan with 44 DSBs (Fig. 3A) [95, 96]. Apart from the above, other means of optimization, including replacement of sulfhydryl oxidases from other sources [97], inversion or development of a periplasmic transmembrane disulfide bond-forming enzyme DsbB [98, 99], were also used to improve the efficiency and capacity of DSB formation.

In addition to the formation of DSBs, the efficiency of other PTMs also affects the functional activity of RPs, such as phosphorylation, acetylation (Fig. 3B), glycosylation and many other modifications that are often found in mAbs and functional proteins [100–102]. Among them, glycosylation is one of the most abundant and complex PTMs [103]. By linking monosaccharides, oligosaccharides or polysaccharides to proteins, the variety of protein functional activities is greatly expanded. Currently, over 70% of therapeutic proteins are modified by glycosylation, and precision glycosylation can effectively enhance the use of glycoproteins in the medical industry [102]. Compared to eukaryotes, *E. coli* does not have a natural mechanism for glycosylation of encoded proteins. Therefore, it can be used as a suitable chassis cell to develop bottom-up glycoengineering for different types of glycoproteins [104]. The first N-glycosylation expression system was successfully developed in *E. coli* by introducing genes related to N-glycosylation of *Campylobacter jejuni*, opening the curtain on the glycoprotein synthesis in *E. coli* [105] (Fig. 3C). Over the last two decades, many efforts have conferred the potential to produce a wide range of N/O-glycoproteins from *E. coli* or cell-free extracts, including optimization of glycosyltransferase substrate identification and orthogonality [102, 106–108], exploration of glycosylase function from multiple sources [107–109] and optimization of host environment, metabolic pathways and culture conditions [110–113]. Based on these studies, a variety of medically relevant products are in production and in the clinical phase, such as recombinant vaccine exotoxin A [114], therapeutic protein O-glycosylated interferon-α2b [115] and N-glycosylated mannose3-N-acetylglucosamine2 [116]. In a similar way to DSB, the glycosylation process in the above systems is mostly completed in the periplasmic space. In recent years, several studies have identified cytoplasmic glycosylation systems in various bacteria, laying the foundation for the development of novel glycosylation systems in *E. coli* [117–119]. Among them, the asparagine (N)-glucosyltransferase from Actinobacillus pleuropneumoniae (ApNGT) can be actively expressed in the *E. coli* cytoplasm and transfer glucose residues to the naturally N-terminal glycosylation site of the protein (e.g. recombinant human EPO) [117]. Based on this discovery, Tytgat et al. [120] developed an N-glycosylation system in *E. coli* cytoplasm. Using ApNGT in combination with various oligosaccharide synthesis pathways (e.g. human milk oligosaccharides and glycosphingolipids), glycosylation modifications of various glycoproteins (glycoconjugate vaccines and multivalent glycopolymers) have been achieved. Surprisingly, the system can complete the glycosylation of megadalton protein assemblies, which can be used as customized carriers for delivery of drugs and vaccines.

It is worth mentioning that the orthogonality of ncAAs with specific codons can be used to introduce various types of modified amino acids more directly and precisely. Park et al. [121] successfully introduced phosphorylated serine residues into RPs at specific sites by orthogonal pairing of SepRS/tRNASep (Fig. 3D). Similarly, phosphor-threonine [122] and phospho-tyrosine [123] were utilized for RP modification. In addition to phosphorylation, acetylation, methylation and ubiquitination have been successfully introduced into various RPs [124]. In conclusion, the introduction of PTMs using ncAAs has the potential to once again make *E. coli* a "star host" for biopharmaceuticals.

### Elimination of inclusion bodies

In addition to limited PTMs, a variety of factors such as misfolding, low solubility, and host burden also contribute to IB formation. Three strategies are usually used to solve the problems: (i) enhancing solubility; (ii) improving correct folding efficiency; (iii) optimizing the appropriate expression intensity. Among them, the relevant aspects of (iii) have been described above.

The use of peptide tags is the most direct and effective means to enhance the solubility of RPs. Common tags include maltose binding protein (MBP), glutathione-S-transferase (GST), carbohydrate-binding module (CBM), thioredoxin, and NusA, which have been reviewed by Ki et al. [125]. Notably, a novel CBM (CBM66) was shown to have a pro-solubilizing effect on several types of RPs and to increase production titer [126]. For example, the combination of poly (ethylene terephthalate) hydrolase and CBM resulted in a 3.7-fold improvement compared to the other commercial labels (MBP and GST), without affecting protein bioactivity. However, if the molecular weight of the peptide tag is close to or larger than that of the RP, it will override the solubility of the RP itself. Furthermore, the subsequent label removal can negatively affect the solubility and stability of RPs. Conversely, the use of peptide tags with smaller molecular weights allows more reliable evaluation and optimization of the solubility of RPs. In recent years, a variety of low-molecular-weight protein tags have contributed to the solubilization and yield enhancement of various RPs, including the NEXT tag [127], low-molecular-weight protamine [128], and 6HFh8 [129]. Kim et al. utilized 6HFh8 [129] to express a variety of growth factor proteins. Among them, 6HFh8-aFGF and 6HFh8-VEGF165 obtained high respective yields of 9.7 and 3.4 g/L in a 5-L batch supplement fermentation, with a purity of more than 99%. The removal of the small peptide tags does not significantly affect the solubility and functional activity, which is suitable for the purification of small RPs.

Molecular chaperones are a class of auxiliary proteins that facilitate the folding and assembly of peptide structures, ensuring proper folding and preventing the aggregation of newly translated peptides [130]. *E. coli* possesses several molecular chaperone systems, such as GroES/EL and DnaK-DnaJ-GrpE, all with different functions [131]. Among them, DnaK-DnaJ-GrpE not only helps correctly fold newly translated peptides, but also functions during co- and post-translational modification. By contrast, the GroES/EL system associates with peptides only post-translationally, powering the repair of misfolded proteins [127]. It is easy to understand that the folding efficiency can be effectively enhanced by overexpression of molecular chaperones, which is usually done in three combinations: GroES/GroEL, DnaK-DnaJ-GrpE, and co-expression. However, co-expression is usually not better than expressing a single factor, and only some chaperones can have a beneficial effect on protein folding [132]. Huang et al. [133] expressed distinct combinations of molecular chaperones to enhance the solubility and activity of polyunsaturated fatty acid isomerase (PAI). The results showed that overexpression of GroES/EL increased the solubility of PAI from 29 to 97% and improved its specific activity by 57.8%. By contrast, the co-expression of DnaK-DnaJ-GrpE or GroES/EL had a weakening effect, resulting in only an 11.9% increase in activity.

## Conclusion and outlook

**Fig. 4:**
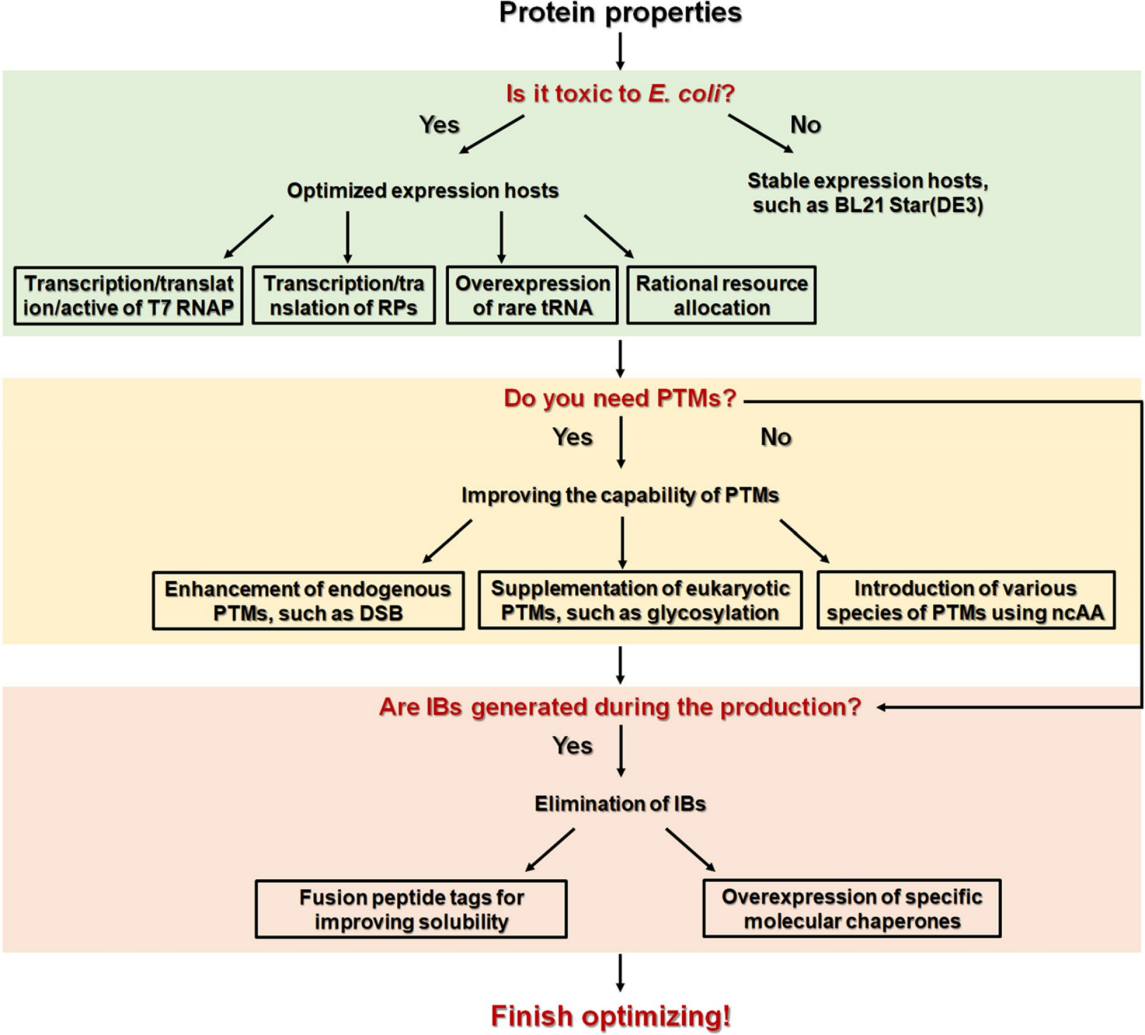
The routine workflow for expression optimization based on protein properties

Different types of RPs from different origins have highly specific characteristics, and there can be no single optimization strategy that applies to all proteins. This review summarizes the recently developed optimization strategies from the two major aspects of alleviating the host burden and optimizing functional activity, which helps researchers quickly select an appropriate expression strategy for their protein of interest (Table 1, Fig. 4). Encouragingly, with the continued development of synthetic biology, systems biology, and various gene editing tools, it is becoming less difficult to rapidly develop a customized host. Multiple in vivo mutagenesis strategies facilitate adaptive laboratory evolution for rapid screening of strongly tolerant expression hosts, including DNA replication proteins, RNAP and T7 RNAP fused with base deaminases [134–137]. Construction of artificial organelles allows for *E. coli* compartmentalization, which has the potential to accomplish precise PTMs [138, 139]. In addition, researchers are updating the BL21(DE3) genome annotation, as well as combining mathematical modeling, statistical analysis, and computer aided design to achieve precise optimization [140, 141]. In conclusion, we have reason to believe that *E. coli* will remain one of the brightest stars among RP production hosts.

**Table 1:** Application of strategies to enhance recombinant protein production in *E.Coli*

Type of optimization	Optimization strategy	Specific optimization method	Application	References
Regulating the RPs expression level	T7 RNAP transcriptional regulation	The promoter lacUV5 and lac were recombined to yield the promoter variants lac-1G. (A at the + 1 position was changed to G)	Over ten autolysis proteins were increased in yield. GDH activity was increased from 37.5 to 452.0 U/ml at 43 h	[20]
	T7 RNAP transcriptional regulation	Mutant LacI (V192F, does not bind IPTG) prevents leaky expression of T7 RNAP and dynamically regulates transcript levels	Three membrane proteins were increased in yield. *E. coli* cytosine transporter protein increased 4.5-fold at 6 h	[31]
	T7 RNAP translational regulation	Rapid screening of suitable expression hosts using the base editor and CRISPR/Cas9 to construct an T7 RNAP RBS library	The expression level of a target gene in the variant strain library ranged from 28 to 220% of the parental strain. The GDH expression exhibited a 298-fold increase	[35]
	T7 RNAP activity regulation	Single amino acid mutation in T7 RNAP (A102D), which reduced the ability to bind to the P_T7_ and decreased the RP production rate	Seven membrane proteins with varying degrees of production improvement (data not mentioned in the article)	[37]
	T7 RNAP activity regulation	T7 RNAP was split into two fragments and expressed in tandem with a light-sensitive dimerization domain, which are functional activity under blue light	The expression intensity of mCheery was increased 80-fold under blue light irradiation compared to dark conditions	[43]
	Plasmid expression regulation	Construction of an ITR library for pET-28a using degenerate primers and MEGAWHOP PCR, and rapid screening of mutants for strong transporter-competent signal peptides by β-lactamase	The multiple types of RPs (mAbs and human growth hormone (hGH)) were increased in yield. The yield of hGH reached 2.56 mg/L, a more than threefold increase	[51]
	Plasmid expression regulation	Regulation of ColE1 plasmid replication-associated gene expression intensity using CRISPRi and the inducible promoter (P_tet_)	The Plasmid libraries containing 1194 different copy numbers, increasing the yield of violacein	[53]
Balancing or decoupling the growth and production	Nutrient supplementation	Amino acids were supplemented according to the level of demand (refer to Sect. 3.1 for specific types and added content)	The production of pramlintide increased by 40% (protein concentration of 3.09 ± 0.12 g/L)	[24]
	Blocking the phosphotransferase system	Blocking the phosphotransferase system (PTS) by knockout ptsG or integration ptsG mutants. can effectively reduce the rate of glucose uptake and decrease the production of acetate	A variety of RPs were increased in yield, including eGFP (increased by 282%), vaccines (increased by 3.5-fold) and glutamate dehydrogenase (increased by 14.84%)	[61–63]
	Blocking the cellular stress response	Transcriptome analysis to identify genes associated with cellular stress response and knockout them. The blocking of CSR alleviates the down-regulated expression of a variety of growth-essential genes	The double knockout mutant BW25113ΔelaA + ΔcysW (DKO) had the highest activity in asparaginase production with 70.3 units/ml	[64]
	Rare codon supplementation	Integrating six of the least abundant tRNA genes into the BL21(DE3) chromosome behind a ribosomal manipulator for expression	Increased yields of eight proteins, which have different lengths and rare codon contents	[13]
	Blocking host growth	Induction of serine recombinase expression and knockout oriC (replication start gene) at late growth stages, blocking host growth	fivefold increase in RFP production	[80]
	Inhibiting host growth	constructing a sgRNA library targeting growth-related genes, and 1332 different sgRNAs were screened to reduce host growth and increase GFP accumulation	GFP production increased more than fivefold when sibB/ibsB was inhibited	[82]
	RPs Expression using o-ribosome	Construction of a specific 16S rRNA recognizing the RPs RBS site for translation	The yield of RPs is 6.3-fold higher than that of the wild type	[84]
Enhancing protein activity	Enhancing transit capacity to the periplasmic space	Enhanced TAT translocation pathway by overexpression of TatABC membrane protein. Meanwhile, the TAT translocation pathway was exploited by the signal peptide TorA fusion RPs	The yield of purified periplasmic hGH are 5.4 g/L	[92]
	Enhancing disulfide bond formation	a host called CyDisCo was developed by overexpressing sulfhydryl oxidase from the yeast mitochondria and disulfide bond isomerase from human cells	Efficient expression of DSB-rich RPs, including antibodies and therapeutic proteins. perlecan, a protein with 44 DSBs, can also be efficiently generated using this host	[95, 96]
	Efficient glycosylation in the cytoplasm	Combination of ApNGT overexpression and various oligosaccharide synthesis pathways for cytoplasmic N-glycosylation	The various glycoproteins have been achieved,including glycoconjugate vaccines, multivalent glycopolymers and megadalton protein assemblies	[120]
	Fusion solubilisation tag improves protein solubility	a variety of low-molecular-weight protein tags have contributed to the solubilization and yield enhancement of various RPs, requiring only fusion expression with recombinant proteins	The two growth factor fusions 6HFh8 tag can substantially increase the yield, reaching 9.7 and 3.4 g/L respectively	[129]
	Overexpression of molecular chaperones	Select appropriate molecular chaperones for overexpression to improve folding efficiency, including GroES/GroEL, DnaK-DnaJ-GrpE, and co-expression	The overexpression of GroES/EL increased the solubility of polyunsaturated fatty acid isomerase from 29 to 97% and improved its specific activity by 57.8%	[133]
